# Ovarian Hyperthecosis Presenting With Postmenopausal Virilization and New-Onset Type 2 Diabetes

**DOI:** 10.1210/jcemcr/luaf118

**Published:** 2025-05-29

**Authors:** Jeremy A Knott, Jack Morris

**Affiliations:** Department of Endocrinology, Wollongong Hospital, Wollongong, NSW 2500, Australia; St George and Sutherland Clinical School, University of New South Wales, Sydney, NSW 2217, Australia; Department of Endocrinology, Shoalhaven District Memorial Hospital ISLHD, Nowra, NSW 2541, Australia; Graduate School of Medicine, University of Wollongong, Wollongong, NSW 2522, Australia

**Keywords:** postmenopausal virilization, ovarian hyperthecosis, hyperandrogenism, hirsutism, metabolic syndrome

## Abstract

Postmenopausal virilization accompanied by insulin resistance is rare and may present as a diagnostic challenge. Ovarian hyperthecosis is characterized by ovarian stromal cell proliferation, leading to androgen excess and associated insulin resistance. Here, we present a case of a 58-year-old postmenopausal woman with new onset virilization and associated type 2 diabetes due to ovarian hyperthecosis, who was successfully treated with bilateral oophorectomy, resulting in normalization of hyperandrogenism and improvement in glycemic control along with metformin and lifestyle advice. This case underscores the importance of recognizing ovarian hyperthecosis as a differential diagnosis when assessing new onset virilization and metabolic disturbance in postmenopausal women.

## Introduction

Virilization in postmenopausal women is uncommon and requires a comprehensive approach that combines clinical assessment, laboratory evaluation, imaging and, in certain cases histopathology to make an accurate diagnosis. Ovarian hyperthecosis is a condition characterized by the proliferation of luteinized ovarian stromal theca cells, resulting in increased androgen production. This results in substantial biochemical and clinical manifestations of progressive hirsutism and virilization. Ovarian hyperthecosis is strongly associated with insulin resistance and metabolic syndrome [1]. Here, we report a case of ovarian hyperthecosis in a postmenopausal woman presenting with virilization and new-onset type 2 diabetes, which was successfully treated with bilateral oophorectomy in conjunction with metformin.

## Case Presentation

**Figure 1.**
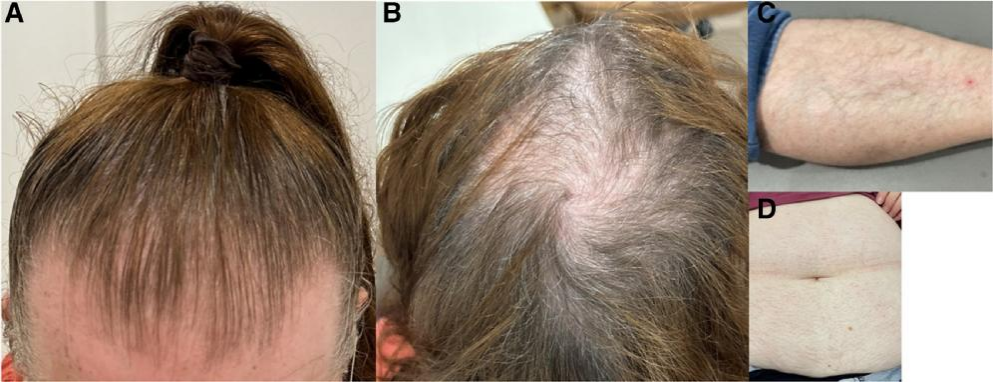
Clinical features of hirsutism with A, hair thinning; B, male-pattern baldness; and C and D, coarse hair growth with evidence of central obesity.

A 58-year-old postmenopausal White woman presented with a 1-year history of progressive features of hirsutism, including coarse facial hair, male-pattern baldness, and hair thinning (Fig. 1). She also described a 6-month history of glucotoxic symptoms with polyuria and polydipsia and approximately 4 kilograms (8.8 pounds) of weight gain and was subsequently diagnosed with type 2 diabetes with a glycated hemoglobin A_1c_ (HbA_1c_) of 9.2% (77 mmol/mol) (reference, <6.5%; 48 mmol/mol) and fasting serum glucose of 9 mmol/L (162.2 mg/dL) (reference, 3.6-6 mmol/L; 64.9-108.1 mg/dL). She had no family history of diabetes, and prior to the development of her hirsutism, her fasting serum glucose was normal at 5.4 mmol/L (97.33 mg/dL) (reference, 3.6-6 mmol/L; 64.9-108.1 mg/dL). Her other medical history included hypertension managed with telmisartan 40 mg daily, hypercholesterolemia managed with atorvastatin 10 mg daily, and obesity (body mass index [BMI] 37.5).

On physical examination, she had hair thinning, male-pattern baldness, and coarse facial and body hair (modified Ferriman-Gallwey score was 14/36) [2]. Her weight was 95 kilograms (209.4 pounds) with evidence of central obesity. There were no other clinical signs of insulin resistance, including acanthosis nigricans or skin tags, and she was not cushingoid. Gynecological examination revealed clitoromegaly.

## Diagnostic Assessment

Laboratory investigations revealed markedly elevated androgen levels: total testosterone 9.9 nmol/L (285.3 ng/dL) (reference, 0.2-1.1 nmol/L; 5.7-31.7 ng/dL) measured by liquid chromatography–tandem mass spectrometry assay, androstenedione 18.4 nmol/L (527.2 ng/dL) (reference, 1-13 nmol/L; 28.6-372.5 ng/dL), and a free testosterone 135 pmol/L (38.8 pg/mL) (reference, 1-22 pmol/L; 0.3-6.3 pg/mL) calculated using the Vermuelen equation. Gonadotrophins were not suppressed: luteinizing hormone 16.3 IU/L (reference, 5-62 IU/L) and follicle-stimulating hormone 21.7 IU/L (reference, 20-140 IU/L). Progesterone was less than 1.6 nmol/L (0.5 ng/mL) (reference, 0.3-0.6 nmol/L; 0.1-0.2 ng/mL), consistent with postmenopausal levels. Estradiol was elevated for a postmenopausal woman at 118 pmol/L (32.1 pg/mL) (reference, <103 pmol/L; <28.1 pg/mL), considered to be due to peripheral testosterone aromatization. Sex hormone–binding globulin (SHBG) was 57 nmol/L (6.4 µg/mL) (reference, 16-120 nmol/L; 1.8-13.5 µg/mL) and dehydroepiandrosterone sulfate (DHEA-S) was 4.6 µmol/L (170.4 µg/dL) (reference, 1-7 µmol/L; 37-259.3 µg/dL) within normal ranges. A Cushing syndrome screen was negative with a normal 24-hour urinary free cortisol of 44 nmol/24 hour (1.6 µg/24 hour) (reference, <166 nmol/24 hour; 6 µg/24 hour), midnight salivary cortisol of less than 3 nmol/L (0.1 µg/dL) (reference, <8 nmol/L; 0.3 µg/dL), and 1-mg dexamethasone suppression test cortisol of less than 28 mmol/L (<1.0 µg/dL) (reference, <50 mmol/L; <1.8 µg/dL). Congenital adrenal hyperplasia was excluded with a normal 17-hydroxyprogesterone of 2.2 nmol/L (72.7 ng/dL) (reference, <1.3 nmol/L; 43.0 ng/dL), noting values up to 4 nmol/L (132.2 ng/dL) are common in postmenopausal women.

**Figure 2.**
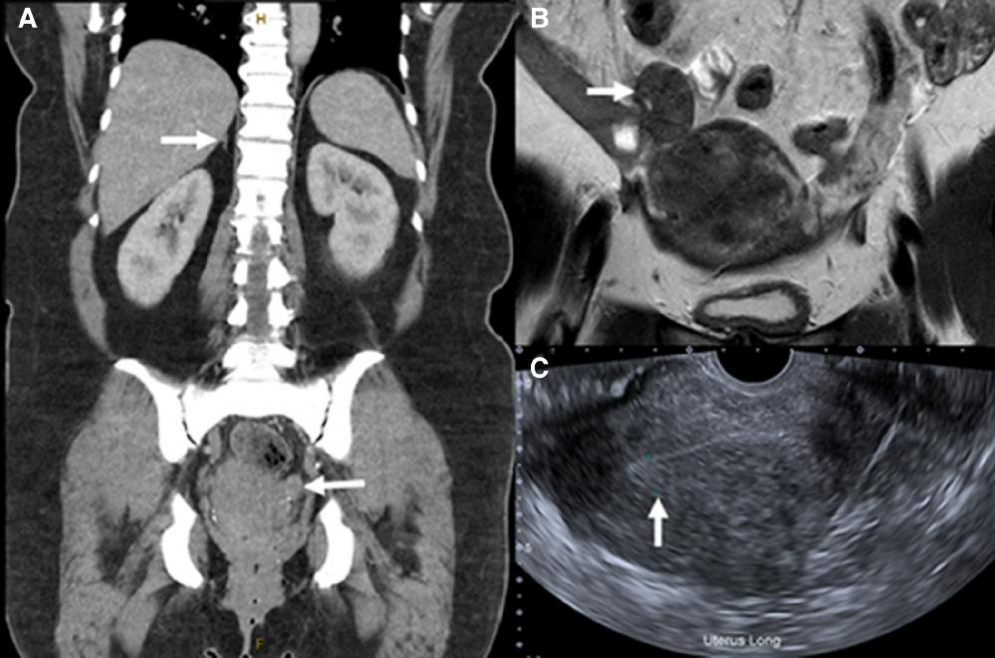
Abdominal A, computed tomography (CT) and B, magnetic resonance imaging (MRI) scans were inconclusive, with no obvious adrenal or ovarian masses. A, The coronal CT view shows the left ovary (arrow) with no masses evident and the right adrenal gland (arrow) with normal contour. B, The coronal MRI view shows the right ovary (arrow) without any identifiable lesion. C, A pelvic ultrasound scan was also inconclusive with no obvious ovarian masses but demonstrated uterine fibroids and endometrial thickening (arrow).

Further imaging studies, including abdominal computed tomography (CT) and magnetic resonance imaging (MRI), were also inconclusive, with no obvious adrenal or ovarian masses identified. Pelvic ultrasound revealed fibroids with endometrial thickening and her ovaries were unable to be visualized (Fig. 2). Gonadal vein sampling was considered, but due to geographical isolation, the patient did not have access to a center with expertise.

## Treatment

**Figure 3.**
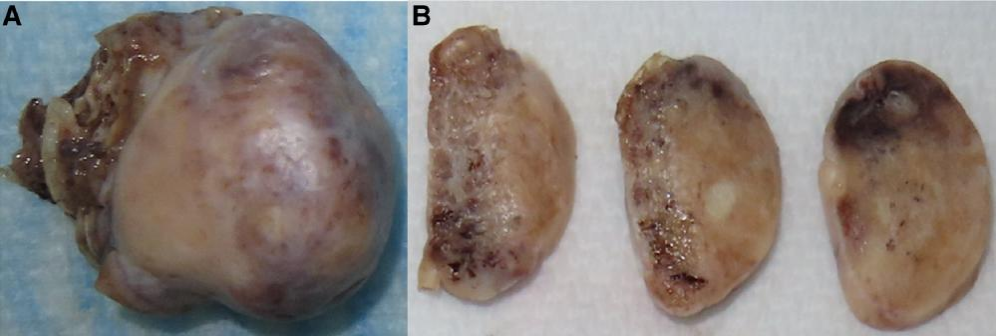
Macroscopic images of resected A, ovary with B, cut slices.

**Figure 4.**
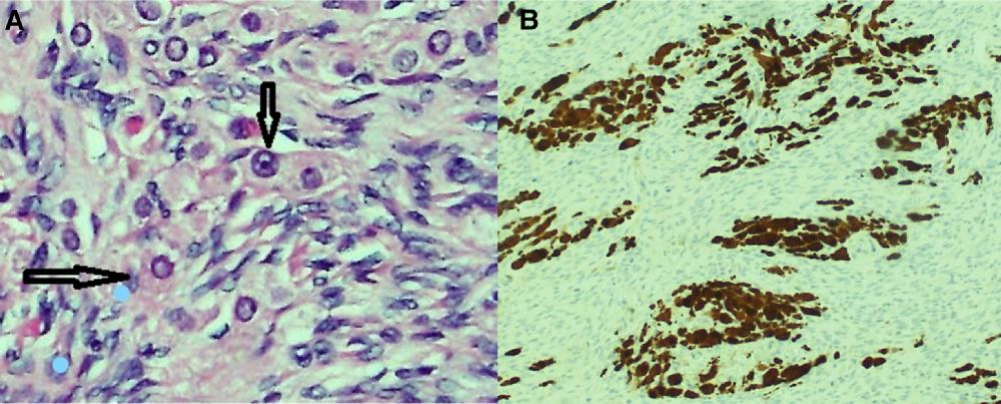
A, Luteinized ovarian theca cells (black arrow) with an eosinophilic, nonvacuolated cytoplasm and regular, round nuclei with prominent nucleoli on hematoxylin-eosin staining. B, Storiform proliferation of luteinized ovarian stromal cells on calretinin staining.

Repeat biochemical evaluation revealed persistent hyperandrogenism and after multidisciplinary team discussion, the patient underwent a diagnostic and therapeutic laparoscopic hysterectomy and bilateral oophorectomy. Macroscopically, the ovaries appeared large for a postmenopausal woman—measuring 39 × 27 × 18 mm (left) and 38 × 27 × 20 mm (right)—and demonstrated a nodular external surface (Fig. 3). Histopathology confirmed ovarian hyperthecosis with luteinized theca cells within the ovarian stroma and no evidence of neoplasia (Fig. 4). For her newly diagnosed type 2 diabetes, she was commenced on metformin 1000 mg daily and dietary and lifestyle advice was provided at the time of presentation.

## Outcome and Follow-up

Postoperatively, androgens normalized; serum androstenedione measured 5.8 nmol/L (166.2 ng/dL) (reference, 1-13 nmol/L; 28.6-372.5 ng/dL) and total testosterone measured 0.9 nmol/L (25.9 ng/dL) (reference, 0.2-1.1 nmol/L; 5.7-31.7 ng/dL). Estradiol was undetectable at less than 88 pmol/L (<24.0 pg/mL) (reference, <103 pmol/L; <28.1 pg/mL), and fasting serum glucose normalized to 5.3 mmol/L (95.5 mg/dL) (reference, 3.6-6 mmol/L; 64.9-108.1 mg/dL) with no significant change in her weight, suggesting an effect of androgen excess on insulin sensitivity. The patient also developed transient menopausal vasomotor symptoms, which was presumed due to reduced aromatization of excess testosterone to estrogen and lowering of estradiol levels. Follow-up visits 6 months postoperatively revealed improved glycemic control with an HbA_1c_ of 6.5% (48 mmol/mol) (reference, <6.5%; <48 mmol/mol) and fasting serum glucose of 5.2 mmol/L (93.7 mg/dL) (reference, 3.6-6 mmol/L; 64.9-108.1 mg/dL), noting this was in conjunction with dietary and lifestyle advice as well as metformin 1000 mg daily. The patient lost approximately 4 kilograms (8.8 pounds) in weight with an improved BMI of 35.5. There was an improvement in facial hair growth, but she had persistent male-pattern baldness. Her blood pressure was maintained with regular telmisartan 40 mg daily, and her hypercholesterolemia with atorvastatin 10 mg daily.

## Discussion

This case underscores the importance of maintaining a high index of suspicion for ovarian hyperthecosis in postmenopausal women presenting with new-onset virilization and metabolic syndrome. Making a diagnosis can be challenging, as conventional imaging modalities including pelvic ultrasound, CT, and MRI may fail to detect the underlying pathology, as demonstrated in our patient. Furthermore, serological testing revealed that gonadotrophins were not suppressed despite elevated testosterone and estradiol levels. This inappropriately normal pattern has been described in ovarian hyperthecosis [3], in which ongoing ovarian steroidogenesis may blunt the expected postmenopausal rise in gonadotrophins. Definitive diagnosis often relies on histopathological examination following surgery, reinforcing the need for clinical vigilance when imaging is inconclusive.

Ovarian hyperthecosis is commonly associated with insulin resistance and hyperinsulinemia, contributing to an increased risk of metabolic dysfunction [3]. Hyperandrogenism has been shown to adversely affect lipid metabolism by promoting visceral adiposity and enhancing proinflammatory pathways, thereby exacerbating insulin resistance [4]. Moreover, hyperinsulinemia itself promotes increased production of androgens, as both insulin and insulin-like growth factor 1 receptors are present in the ovary to induce stromal luteinization and directly stimulate theca cells to produce androgens, acting in a synergistic cycle to contribute to increased insulin resistance [5]. Additionally, hyperinsulinemia suppresses hepatic production of SHBG, further increasing levels of circulating free testosterone and amplifying the metabolic risks associated with androgen excess [6, 7]. Clinical studies of antiandrogenic treatment has been shown to partially improve insulin resistance [8], highlighting the role of androgen excess on the impairment of insulin action. Surgical management of ovarian hyperthecosis with bilateral oophorectomy normalizes hyperandrogenism and may contribute to subsequent improvement in glycemic control in women with diabetes [9, 10].

While the observed improvement in HbA_1c_ from pre surgery 9.2% (77 mmol/mol) to 6.5% (48 mmol/mol) at 6 months post surgery is notable, this degree of glycemic improvement may reflect the combined effects of lifestyle modification, metformin therapy (1000 mg daily), and surgical intervention. Notably, a double-blind trial evaluating metformin at a dose of 2000 mg daily demonstrated an approximate 2% reduction in HbA_1c_ over 3 months in individuals with higher baseline HbA_1c_ levels (mean 9.9%), highlighting the significant glucose-lowering potential of pharmacotherapy in this context [11]. In a randomized controlled trial of 35 healthy postmenopausal women without diabetes, those receiving metformin experienced a 19% reduction in free testosterone compared with placebo, alongside expected improvements in insulin sensitivity [7]. This highlights the additive role of pharmacological interventions, in conjunction with lifestyle measures, in optimizing metabolic outcomes for ovarian hyperthecosis.

Alongside increased metabolic risk, patients with ovarian hyperthecosis are also at increased risk of endometrial hyperplasia and carcinoma, which may arise from peripheral aromatization of androgens to estrogens in the setting of hyperandrogenism [12, 13]. Endometrial thickening observed on pelvic ultrasound in our patient was suspected to be due to unopposed estrogen exposure from aromatization of free testosterone. It highlights the need for prompt diagnosis and management of ovarian hyperthecosis to reduce the risk of further potential complications [7].

Our case emphasizes the importance of considering ovarian pathology in the evaluation of postmenopausal virilization and insulin resistance. The normalization of androgen levels following bilateral oophorectomy may have contributed to improved glycemic control, underscoring the role of androgen excess in insulin resistance, in conjunction with pharmacotherapy and lifestyle measures. This report highlights the potential for significant clinical improvement through a multidisciplinary approach, including surgical, medical, and lifestyle advice, for managing ovarian hyperthecosis and its metabolic complications.

## Learning Points

- It is important to consider ovarian hyperthecosis early in the differential diagnosis of postmenopausal virilization, particularly in the presence of insulin resistance and metabolic syndrome.
- Although imaging findings are often nonspecific, histopathology remains crucial for confirming ovarian hyperthecosis.
- Ovarian hyperthecosis is a treatable cause of androgen excess, and surgical resection may lead to significant clinical improvements in conjunction with pharmacotherapy.

## Contributors

All authors made individual contributions to authorship. J.M. was involved in the diagnosis and management of this patient. J.K. was involved in manuscript write-up and submission.

## Data Availability

Some or all data sets generated during and/or analyzed during the current study are not publicly available but are available from the corresponding author on reasonable request.

## Abbreviations

BMI: body mass index
CT: computed tomography
HbA_1c_: glycated hemoglobin A_1c_
MRI: magnetic resonance imaging
SHBG: sex hormone–binding globulin
